# Opioid-free anesthesia with lidocaine for improved postoperative recovery in hysteroscopy: a randomized controlled trial

**DOI:** 10.1186/s12871-023-02152-7

**Published:** 2023-06-03

**Authors:** NH Cha, Y Hu, GH Zhu, X Long, JJ Jiang, Yuan Gong

**Affiliations:** Institute of Anesthesiology and Critical Care Medicine, Three Gorges University & Yichang Central People’s Hospital, No. 183 Yiling Avenue, Wujiagang District, 443000 Yichang City, Hubei China

**Keywords:** Opioid-free anesthesia, Opioids, Postoperative nausea and vomiting, Quality of recovery (QoR)

## Abstract

**Background:**

Anesthesia with opioids negatively affects patients’ quality of recovery. Opioid-free anesthesia attempts to avoid these effects. This study aimed to evaluate the effect of opioid-free anesthesia on the quality of recovery, using lidocaine on patients undergoing hysteroscopy.

**Methods:**

A parallel-group, randomized, double-blind, controlled trial was conducted in Yichang Central Peoples’ Hospital, Hubei Province, China, from January to April, 2022. We included 90 female patients (age: 18–65 years, American Society of Anesthesiologists Physical Status Class I–II) scheduled for elective hysteroscopy, 45 of whom received lidocaine (Group L), and 45 received sufentanil (Group S). Patients were randomly allocated to receive either lidocaine or sufentanil perioperatively. The primary outcome was the quality of postoperative recovery, which was assessed using the QoR-40 questionnaire (a patient-reported outcome questionnaire measuring the quality of recovery after surgery).

**Results:**

The two groups were similar in age, American Society of Anesthesiology physical status, height, weight, body mass index, and surgical duration. The QoR scores were significantly higher in Group L than Group S. The incidence of postoperative nausea and vomiting, as well as the time to extubation were significantly lower in Group L than Group S.

**Conclusion:**

Opioid-free anesthesia with lidocaine achieves a better quality of recovery, faster recovery, and a shorter time to extubation than general anesthesia with sufentanil.

**Trial registration:**

The trial was registered on January 15, 2022 in the Chinese Clinical Trial Registry (http://www.chictr.org.cn/showprojen.aspx?proj=149386), registration number ChiCTR2200055623.(15/01/2022).

**Supplementary Information:**

The online version contains supplementary material available at 10.1186/s12871-023-02152-7.

## Background

Hysteroscopy is traditionally performed under general anesthesia for pain management. Opioids are an essential part of anesthesia for their antinociceptive effects; however, they are associated with side effects, including altered mental status, hyperalgesia, nausea, and vomiting, which have a negative effect on patients’ experience [1, 2].

Opioid-free anesthesia (OFA) is a multimodal approach to inducing anesthesia without opioids. The drugs used for OFA include, among others, local anesthetics. Lidocaine is a local amide anesthetic that blocks sodium channels and N-methyl-D-aspartate receptors [3]. The rationale for OFA is to avoid the side effects of opioids, which can impact the patient’s postoperative outcome [4]. In other words, better recovery can be expected in hysteroscopic patients undergoing OFA. Recent developments in the anesthesia field are ensuring faster recovery from anesthesia, and optimizing surgical patients’ subjective postoperative experience. Studies have demonstrated that patient-reported outcome measures can improve patient experience after surgery [5]. QoR-40 is a patient-reported outcome questionnaire that measures the quality of recovery after surgery; the results are a useful end-point in perioperative clinical studies [6].

The use of OFA is highly controversial, with limited evidence on the potential benefits; one study showed severe side effects [7, 8]. This study aimed to compare the quality of recovery, recovery time, and risk of side effects of between OFA with lidocaine and general anesthesia with opioids in patients undergoing ambulatory hysteroscopy.,

## Methods

### Study design

This was a parallel-group, randomized, double-blind, controlled trial. Ethical approval for this study (HEC-KYJJ-2021-145-01) was provided by the Institutional Review Board of Yichang Central People’s Hospital, Wujiagang District, Yichang City, Hubei Province (contact: Yan Kejun) on January 7, 2022. The trial was registered on January 15, 2022 in the Chinese Clinical Trial Registry (http://www.chictr.org.cn/showprojen.aspx?proj=149386), registration number ChiCTR2200055623. The study was conducted from January 25 to April 30, 2022 at Yichang Central People’s Hospital. All patients provided written informed consent. The patients were randomly assigned to two groups in a 1:1 ratio by computer-generated randomization. One anesthesiologist, who was blinded to the study, generated the random allocation sequence and assigned each patient a patient code.

### Patients

We included female patients (age: 18–65 years, American Society of Anesthesiologists Physical Status Class I–II) scheduled for elective hysteroscopy. Patients were divided into two groups: Group S, who received general anesthesia (sufentanil, Yichang Renfu Pharmaceutical Co. LTD, Yichang city, China) and Group L, who received OFA (lidocaine, Anhui Changjiang Pharmaceutical Co. LTD, Wuhu City, China). The exclusion criteria were as follows: history of drug abuse, refusal to provide consent, history of psychotropic medication or psychological disorders, treatment using angiotensin-converting enzyme inhibitors, gastro-esophageal reflux, morbid obesity (BMI ≥ 30 kg/m^2^ ), allergy to any of the study drugs, use of medications or nutraceuticals that affect blood pressure (BP) or heart rate (HR), surgical procedure exceeding one hour in duration, unexpected bleeding complications, or repeated laryngeal mask airway insertion attempts.

### Perioperative anesthetic care

Preoperatively, all patients fasted for 8 h and were asked to avoid oral intake of clear fluids for 2 h. Upon entry into the operating room, non-invasive BP, HR, and pulse oxygen saturation were measured, and electrocardiography was performed using a multifunctional monitor (GE Healthcare, Chicago, IL, USA), with 5 min of stabilization after each measurement. A 24-gauge intravenous catheter was subsequently placed into a vein on the dorsum of the hand.

After preoxygenation, either 1.5 mg kg^− 1^ lidocaine or 0.3 µg kg^− 1^ sufentanil was injected intravenously over a 3 s period with activation of the pump, followed by either 1.5 mg kg^− 1^ h^− 1^ lidocaine or 0.9% saline, respectively, at the same rate and volume. The anesthetic nurse who prepared the research treatment solutions and activated the pump was blinded to the study groups. Two minutes after activation, general anesthesia was standardized with 2.0 mg kg^− 1^ propofol (Fresenius Kabi Deutschland GmbH, Homburg, Germany) and 1 mg kg^− 1^ scoline (Xi’an Hanfeng Pharmaceutical Co. LTD, Xi’an City, China). If the patient exhibited no eyelash reflex, an appropriately sized laryngeal mask airway was selected using standard guidelines and the patient was intubated. After intubation without complications, 0.3 mg kg^− 1^ of rocuronium (Zhejiang Xianju Pharmaceutical Co., Ltd., Taizhou City, China) was administered to maintain muscle relaxation. Anesthesia was maintained using 2–3% sevoflurane (Maruishi Pharmaceutical Co., Ltd., Osaka, Japan) and 50% oxygen.

At the end of the surgery, sevoflurane and the infusions were discontinued. Patients were administered 0.04 mg kg^− 1^ neostigmine (Zhejiang Xianju Pharmaceutical Co., Ltd., Taizhou City, China) and 0.02 mg kg^− 1^ atropine (Suicheng Pharmaceutical Co., LTD, Xinzheng City, China) to antagonize any residual neuromuscular blockade, after evaluation with a neuromuscular monitor. During anesthesia, an anesthesiologist blinded to the group assignments oversaw patients’ emergence from anesthesia and graded their postoperative pain. Oral suction was performed immediately after surgery, with the patient still under anesthesia. Extubation was performed when the patient regained consciousness, with confirmation of adequate tidal volume, a regular spontaneous respiratory pattern, and purposeful behavior (eye opening upon request). After extubation, patients were monitored for ≥ 5 min to ensure regular spontaneous respiration, and were subsequently transferred to the post-anesthesia care unit (PACU). Here, patients were monitored with electrocardiography, peripheral pulse oximetry, and non-invasive BP measurements.

Patients were discharged from the PACU when their Steward score reached > 4. Other operative care was provided according to usual clinical practice. Postoperative quality of recovery was investigated using the QoR-40 questionnaire. Postoperative pain was quantified using a 100-mm visual analog scale (VAS), marked from “no pain” to “severe pain.” [9]. If the VAS score was ≥ 30/100 at rest, the attending PACU nurse administered 30 mg kg^− 1^ of intravenous propacetamol. The QoR-40 questionnaire was performed in the ward 24 h postoperatively by an anesthetist that was blinded to the study.

### Primary outcomes

The primary outcome was the quality of recovery 24 h postoperatively as assessed using the QoR-40 questionnaire.

### Secondary outcomes

Secondary outcomes included the incidence of postoperative nausea and vomiting (PONV), and time to extubation. Safety-related outcomes included intraoperative respiratory/cardiovascular complications (laryngospasm, postoperative hypoxemia,arrhythmia) and rescue methods.

### Statistical analysis

The sample size was calculated based on the results of our preliminary experiments and an expected QoR-40 score difference of 10 with 95% power (α = 0.05 and β = 0.1), which indicated that 40 patients were required per group. The QoR-40 scores, patient characteristics (age, height, weight, and body mass index), surgery duration, time to extubation, and time to recovery are expressed as the mean ± standard deviation, and analyzed using student’s t test. The incidence of PONV was expressed as ratio using the chi-square or Fisher exact test; *P* < 0.05 indicated statistical significance. Statistical analyses were performed using GraphPad Prism 8.4.3 (GraphPad Software Inc., San Diego, CA, USA).

### Data Availability

The data associated with the paper are not publicly available but are available from the corresponding author on reasonable request.

## Results

### Patients

We enrolled 90 patients from January 25 to April 30, 2022. No significant intergroup differences were observed in patient age or physical characteristics (Table 1).

**Table 1:** Patient baseline and clinical characteristics

	Group L (n = 45)	Group S (n = 45)	*P* value
Age (y)	38.9 ± 6.3	38.7 ± 7.0	> 0.9999
Height (cm)	159.7 ± 3.9	159.5 ± 3.6	0.9996
Weight (kg)	56.1 ± 8.4	56.3 ± 8.1	0.9992
Body mass index (kg/cm^2^)	22.9 ± 3.7	22.8 ± 3.2	0.9993
Duration of operation (min)	19.3 ± 4.6	18.8 ± 5.5	0.9999
Duration of anesthesia (min)	29.8 ± 6.3	30.0 ± 7.1	> 0.9999
Operation type (diagnostic vs. treatment)	14/31	15/30	0.5164
Postoperative hypoxemia	0	0	
Laryngospasm	0	0	
Arrhythmia	0	0	

Data are presented as either mean ± standard deviation or counts

### Primary outcomes

We discovered significant intergroup differences in the QoR-40 score 24 h postoperatively (*P* ≤ 0.01; Table 2).

**Table 2:** QoR-40 scores

	Group L (n = 45)	Group S (n = 45)	*P* value
QoR-40 scores 24 h postoperatively	177.2 ± 6.2	172.6 ± 5.3	0.0002

Data are expressed as mean ± standard deviation

QoR-40, Quality of recovery-40

### Secondary outcomes

**Fig. 1:**
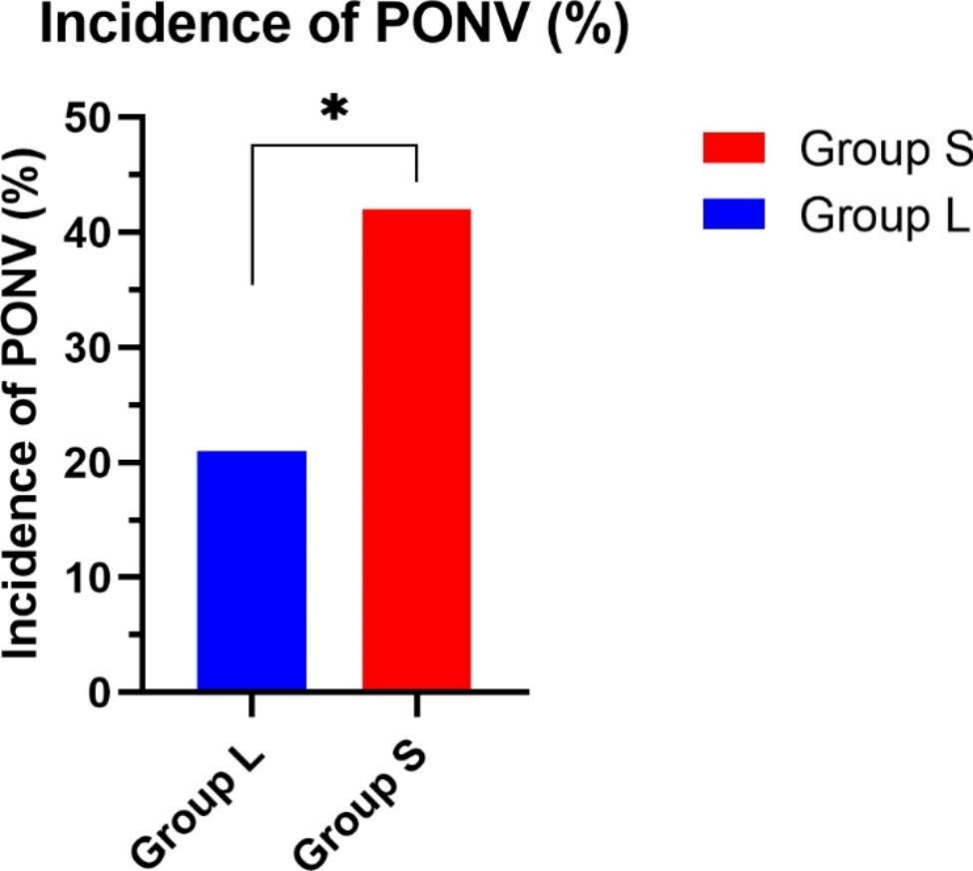
Incidence of postoperative nausea and vomiting (PONV) among the study groups Data are expressed as ratio

We observed a significant intergroup difference in time to extubation (L, 6.93 ± 0.95 min vs. S, 8.33 ± 1.11 min; *P* ≤ 0.01) (Table 3). The PONV incidence was lower in Group L than in Group S (L, 21% vs. S, 42%; *P* ≤ 0.05) (Fig. 1).

**Table 3:** Time of extubation

	Group L (n = 45)	Group S (n = 45)	*P* value
Time of extubation (min)	6.9 ± 1.0	8.3 ± 1.1	< 0.0001

Data are expressed as mean ± standard deviation

We observed no cases of severe complications such as postoperative hypoxemia, laryngospasm,or arrhythmia in this study (Table 1).

## Discussion

The rationale of OFA is to avoid the side-effects associated with opioids on patients’ intra-and post-operative outcomes. Lidocaine is a local anesthetic, with analgesic, anti-hyperalgesic and anti-inflammatory properties. It has traditionally been used as an adjuvant during anesthesia; in this study it was administered intravenously.

Our findings revealed that, compared to general anesthesia with sufentanil, OFA with lidocaine resulted in higher QoR-40 scores in patients who underwent hysteroscopy. Moreover, lidocaine-OFA showed a shorter time to extubation and a lower incidence of PONV compared to sufentanil. Our findings demonstrate that OFA with lidocaine can provide faster and better postoperative recovery after hysteroscopy.

QoR is an important outcome measure of early postoperative health, and it is widely used for assessing patient recovery after anesthesia. The QoR-40 questionnaire includes five items: emotional state, physical comfort, psychological support, physical independence, and pain. The the validity and reliability of the QoR-40 score have been proven [6]. We found statistical intergroup differences in the QoR-40 scores, however, the difference between groups did not reach our pre-set value. This may be due to sampling error in the preliminary experiment. Considering the characteristics of the QoR-40, higher scores reflect better quality of recovery. Our findings demonstrated that OFA with lidocaine allowed better QoR than general anesthesia with sufentanil did, even at 24 h postoperatively; thus, OFA with lidocaine is of clinical importance.

A longer duration of intubation has been demonstrated to result in a higher incidence of mortality [10]. The time of extubation continues to be a variable of considerable importance for patient recovery. We observed a statistically significant intergroup difference in time of extubation. This could be attributed to sufentanil having a high affinity and specificity for opioid receptors, which yields a sedative effect that enhances its effectiveness as an anesthetic [11]. PONV is a common postoperative complication, especially in younger female patients undergoing general anesthesia. It can potentially cause a highly distressing experience and a longer hospital stay [12].. Our study identified a higher rate of PONV in Group S, with a statistically significant intergroup difference (Fig. 1). No severe safety concerns such as postoperative hypoxemia, laryngospasm, or arrhythmia occurred during our study.

This study has several limitations. First, this was a single-center study; therefore, the generalizability of the findings is limited. Second, we included patients who underwent anesthesia using a laryngeal mask airway to maintain the sevoflurane concentration, which reduced the generality of our findings.

## Conclusions

Our trail showed that OFA with lidocaine for hysteroscopy is both safe and effective, and allowed faster and better recovery than that associated with general anesthesia with sufentanil, as assessed using QoR-40 scores. This suggests that OFA with lidocaine can be used in hysteroscopy successfully. It indicated that OFA can be performed in ambulatory surgery smoothly and effectively, and that the prospects for the use of lidocaine in anesthesia warrant further investigation.

## Electronic supplementary material

Below is the link to the electronic supplementary material.

Supplementary Material 1

## Data Availability

The data associated with the paper are not publicly available but are available from the corresponding author (YG) on reasonable request.

## Abbreviations

HR: Heart rate
BP: Blood pressure
OFA: Opioid free anesthesia
PACU: Post-anesthesia care unit
PONV: Postoperative nausea and vomiting
VAS: Visual analog scale

## References

[1] Koepke EJ, Manning EL, Miller TE, Ganesh A, Williams DGA, Manning MW (2018). The rising tide of opioid use and abuse: the role of the anesthesiologist. Perioper Med (Lond).

[2] Kessler ER, Shah M, Gruschkus SK, Raju A (2013). Cost and quality implications of opioid-based postsurgical pain control using administrative claims data from a large health system: opioid-related adverse events and their impact on clinical and economic outcomes. Pharmacotherapy.

[3] Beaussier M, Delbos A, Maurice-Szamburski A, Ecoffey C, Mercadal L (2018). Perioperative use of intravenous lidocaine. Drugs.

[4] Beloeil H (2019). Opioid-free anesthesia. Best Pract Res Clin Anaesthesiol.

[5] Myles PS, Hunt JO, Nightingale CE, Fletcher H, Beh T, Tanil D (1999). Development and psychometric testing of a quality of recovery score after general anesthesia and surgery in adults. Anesth Analg.

[6] Myles PS, Weitkamp B, Jones K, Melick J, Hensen S (2000). Validity and reliability of a postoperative quality of recovery score: the QoR-40. Br J Anaesth.

[7] Hakim KYK, Wahba WZB (2019). Opioid-free total intravenous anesthesia improves postoperative quality of recovery after ambulatory gynecologic laparoscopy. Anesth Essays Res.

[8] Beloeil H, Garot M, Lebuffe G, Gerbaud A, Bila J, Cuvillon P (2021). Balanced opioid-free anesthesia with dexmedetomidine versus balanced anesthesia with remifentanil for major or intermediate noncardiac surgery. Anesthesiology.

[9] Hopkins CS, Buckley CJ, Bush GH (1988). Pain-free injection in infants. Use of a lignocaine-prilocaine cream to prevent pain at intravenous induction of general anaesthesia in 1–5-year-old children. Anaesthesia.

[10] Epstein SK (2009). Weaning from ventilatory support. Curr Opin Crit Care.

[11] Lysakowski C, Dumont L, Pellégrini M, Clergue F, Tassonyi E (2001). Effects of fentanyl, alfentanil, remifentanil and sufentanil on loss of consciousness and bispectral index during propofol induction of anaesthesia. Br J Anaesth.

[12] Gan TJ, Belani KG, Bergese S, Chung F, Diemunsch P, Habib AS et al. Fourth consensus guidelines for the management of postoperative nausea and vomiting. Anesth Analg. 2020;131:411 – 48. Erratum in: Anesth Analg. 2020;131:411 – 48. 10.1213/ANE.0000000000004833 32467512

