# Supplementary material for: Opioid-free anesthesia with lidocaine for improved postoperative recovery in hysteroscopy: a randomized controlled trial
Source: BMC Anesthesiol. 2023 Jun 3;23:192. doi: 10.1186/s12871-023-02152-7 (PMC10239123; doi:10.1186/s12871-023-02152-7)
Supplement: Supplementary file 1 — Supplementary Material 1 [file 12871_2023_2152_MOESM1_ESM.doc]

**Allocation**

**Analysis**

**Follow-Up**

**Flow Diagram**

Assessed for eligibility (n=95 )

Excluded (n= 5 )

♦  Not meeting inclusion criteria (n= 3 )

♦  Declined to participate (n=1 )

♦  Other reasons (n= 1 )

Analysed (n= 45 )
♦ Excluded from analysis (give reasons) (n=0 )

Lost to follow-up (n= 0 )

Discontinued intervention (n= 0 )

Allocated to intervention (n= 45 )

♦ Received allocated intervention (n= 0 )

♦ Did not receive allocated intervention (give reasons) (n= 0)

Lost to follow-up (give reasons) (n= 0 )

Discontinued intervention (give reasons) (n=0 )

Allocated to intervention (n= 45 )

♦ Received allocated intervention (n=0 )

♦ Did not receive allocated intervention (give reasons) (n= 0 )

Analysed (n= 45 )
♦ Excluded from analysis (give reasons) (n=0 )

Randomized (n= 90 )
